# Pharmacokinetic analysis of the chronic administration of the inert gases Xe and Ar using a physiological based model

**DOI:** 10.1186/s13618-015-0029-z

**Published:** 2015-05-29

**Authors:** Ira Katz, Jacqueline Murdock, Marc Palgen, Jan Pype, Georges Caillibotte

**Affiliations:** Medical R&D, Air Liquide Santé International, Centre de Recherche Paris-Saclay, 1, chemin de la Porte des Loges, BP126 - 78354 Jouy en Josas, France; Department of Mechanical Engineering, Lafayette College, Easton, PA 18042 USA

**Keywords:** Pharmacokinetics, Xenon, Argon, Human, Pig, Rat

## Abstract

**Background:**

New gas therapies using inert gases such as xenon and argon are being studied, which would require chronically administered repeating doses. The pharmacokinetics of this type of administration has not been addressed in the literature.

**Methods:**

A physiologically based pharmacokinetics (PBPK) model for humans, pigs, mice, and rats has been developed to investigate the unique aspects of the chronic administration of inert gas therapies. The absorption, distribution, metabolism and excretion (ADME) models are as follows: absorption in all compartments is assumed to be perfusion limited, no metabolism of the gases occurs, and excretion is only the reverse process of absorption through the lungs and exhaled.

**Results:**

The model has shown that there can be a residual dose, equivalent to constant administration, for chronic repeated dosing of xenon in humans. However, this is not necessarily the case for small animals used in pre-clinical studies.

**Conclusions:**

The use of standard pharmacokinetics parameters such as area under the curve would be more appropriate to assess the delivered dose of chronic gas administration than the gas concentration in the delivery system that is typically reported in the scientific literature because species and gas differences can result in very different delivered doses.

## Introduction

Gases with proven or exploratory medical use include oxygen, hydrogen, carbon monoxide, carbon dioxide, hydrogen sulfide, nitric oxide, nitrous oxide, xenon, argon, helium and other noble gases [1–4]. In general, the relatively fast wash-in and wash-out of gases, and their application only for acute treatment (with the exception of oxygen), has made pharmacokinetic (PK) analysis of secondary importance such that it has not been a focus of research or regulation. However, there are notable exceptions. Lockwood and his colleagues have developed experimental techniques and models for the PK analysis of gaseous anesthetics [5, 6]. Filser, Bolt and their colleagues have presented an impressive series of papers on the application of PK in the context of the environmental toxicology of gas pollutants [7–12].

In recent years evidence has been accumulating to indicate that certain inert or noble gases, existing as mono-atomic gases with low chemical reactivity, nevertheless express biological activity. Numerous *in vitro* and *in vivo* experiments have demonstrated intriguing biological effects for xenon, argon and helium, with neuro- and organo-protective properties as the most clinically promising [4, 13]. Extensive research has further revealed some of the underlying mechanisms and include competitive antagonism at the NMDA/AMPA receptor [14], anti-apoptotic properties (inhibition of mitochondrial cytochrome c release) [15], activation of pro-survival signaling pathways (increased expression of Bcl-2/Bcl-xL, inhibition of Bax) [16], MAPK regulation (p38, ERK_1/2_) [17, 18] and potassium ion channels activation (K_ATP_, TREK-1) [19, 20]. The preclinical models suggest potential clinical benefit in indications such as traumatic brain injury, ischemic or hemorrhagic stroke, perinatal hypoxic-ischemic brain injury, coronary artery bypass graft surgery, organ protection during transplantation, chronic pain, and addiction [13, 21–23]. Any clinical benefit, however, would require chronic (or repetitive) administration of inert gases, in contrast to the currently accepted acute, single administration for the induction and maintenance of general anesthesia (e.g. xenon).

From a PK standpoint there are several issues that arise concerning the development and future application of the chronic administration of inert gases. For these reasons we have developed a computational physiological based pharmacokinetic (PBPK) model for inert gases primarily based on the model of Lockwood [5]. The model was used to investigate chronic administration of the noble gases xenon and argon in terms of empirically determined physiological PK parameters (i.e., partition coefficients), species comparisons, and intersubject variability. These issues will inevitably be important in moving from animal models to clinical testing, toxicological testing, the development of delivery devices, and the regulatory purview of gas treatments. One particular aspect of methodology addressed herein is the definition of the dose itself. We use PK variables to define the dose at the site of action, whereas, the dose of a gas treatment is typically given as the concentration of the inhaled gas.

## Methods

A pharmacokinetic model principally following the one described by Lockwood [5] was developed using the Simbiology Toolkit of MATLAB (Mathworks, United States). Simbiology provides a graphical environment and programming tools to model, simulate, and analyze PK applications. Specifically in this case, it allows for simplified development, programming and debugging, of the PK model including the numerical solver of the resulting system of differential equations.

**Fig. 1:**
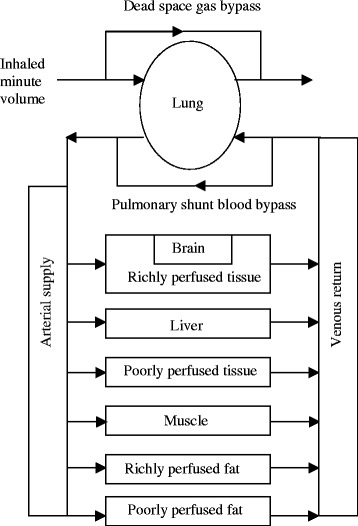
Schematic of the pharmacokinetic model, compartments and gas species flows. The model does not consider the lung tissue per se (except as part of the richly perfused tissue compartment), but the gas volume within it

The model is described by the schematic shown in Fig. 1 and the data listed in Table 1. Regarding Fig. 1, it can be discerned that gas therapy starts in the lung. The model does not consider the lung tissue per se (except as part of the richly perfused tissue compartment), but the gas volume within it. The extreme complexity of the lung and the dynamics of respiration [24–27] are greatly simplified following Lockwood in that the model does not take into account the oscillatory nature of inhalation and exhalation or details of ventilatory distribution. Thus the ventilatory input is the minute volume, inspired tidal volume multiplied by the respiratory rate in breaths per minute. Changes in minute volume can be modeled, but only at a time scale that would include several breaths (minutes) not during a breath (seconds). Morphological complexity of the respiratory tract is accounted for by including a component of dead space; that is gas that never reaches the alveolar gas exchange region of the lung, is considered as a gas bypass of 32.5 % of minute volume (i.e., 162.5 ml of dead space and 500 ml tidal volume). The exhaled gas compartment consists of 90 % alveolar gas and 10 % inhaled gas to account for the complex mixing inherent in real respiration. In terms of pulmonary circulation, 10 % of the cardiac output was shunted past the lung without gas exchange. Thus the equation for transfer from the alveolar gas to the arterial (oxygenated) blood in the lung is

**Table 1:** Physiological data and partition coefficients used for the model

Parameter	Mouse	Rat	Pig	Human (Adult Male)
Body Weight (kg)	0.025	0.25	25	70
Minute Ventilation (l.min^-1^)	0.385	0.18	3.6	7.5
Alveolar Ventilation (l.min^-1^)	0.025	0.117	2.34	4.875
Cardiac Output (l.min^-1^)	0.017	0.083	2.060	6.0
Perfusion per compartment (as a fraction of Cardiac Output)				
Fat (Richly perfused)	0.09	0.09	0.1747	0.04
Fat (Poorly perfused)	NA	NA	NA	0.01
Liver	0.25	0.25	0.3052	0.26
Richly perfused tissue	0.415	0.484	0.1829	0.3303
Poorly perfused tissue	NA	NA	0.0553	0.01
Muscle	0.15	0.15	0.2523	0.24
Brain	0.095	0.026	0.0296	0.093
Volumes (fraction of Body Weight)				
Arterial blood	0.0110	0.0167	0.018	0.0209
Venous blood	0.0331	0.050	0.042	0.0545
Lung blood	0.0049	0.0074	NA	0.00245
Fat (Richly perfused)	0.10	0.07	0.3	0.09
Fat (Poorly perfused)	NA	NA	NA	0.09
Liver	0.055	0.04	0.0294	0.06
Richly perfused tissue	0.0454	0.0497	0.0697	0.0624
Poorly perfused tissue	NA	NA	0.1269	0.24
Muscle	0.66	0.676	0.4	0.44
Brain	0.0046	0.0003	0.004	0.0176
Partition coefficients for Xenon				
Blood:gas	0.207	0.207	0.11	0.14
Fat:blood	6.2802	6.2802	11.8182	9.287
Liver:blood	0.7246	0.7246	1.3636	1.071
Richly perfused tissue:blood	0.6951	0.7229	1.3774	1.071
Poorly perfused tissue:blood	0.7246	0.7246	1.3636	1.071
Muscle:blood	0.7246	0.7246	1.3636	1.071
Brain:blood	1.1233	1.015	1.1233	1.123
Partition coefficients for Argon				
Blood:gas	0.037	0.037	0.037	0.037
Fat:blood	4.1622	4.1622	4.1622	4.162
Liver:blood	0.7539	0.7539	0.7539	0.754
Richly perfused tissue:blood	1.0663	1.0323	1.0506	1.028
Poorly perfused tissue:blood	0.9987	0.9987	0.9987	0.999
Muscle:blood	0.7205	0.7205	0.7205	0.720
Brain:blood	0.6747	0.6747	0.6747	0.675

1 $$ {C}_{arterial\_ lung}=0.9\times \frac{\left(0.9\times AV\times {C}_{inhaled}\right)+\left(CO\times {C}_{venous}\right)}{CO+\frac{0.9\times AV}{P{C}_{blood: gas}}}+0.1\times {C}_{venous} $$

where *AV* is the alveolar ventilation (the inhaled tidal volume less the dead space times the respiratory rate), *C*_*inhaled*_ is the inhaled concentration, *C*_*venous*_ is the concentration of the gas in the venous blood, *CO* is the cardiac output, and *PC*_*blood:gas*_ is the partition coefficient between the gas phase and in solution in the blood. *C*_*inhaled*_ in mol/L is related to the percentage by volume, %gas (equivalent to the molar percentage), by the perfect gas law.

2 $$ \begin{array}{l}{C}_{inhaled}=\frac{P_{total}}{RT}\left(\frac{\%\mathrm{gas}}{100}\right)\\ {}\end{array} $$

where *R* = 8 314.4621 *Pa. L. mol*^− 1^. *K*^− 1^ is the universal perfect gas constant, the temperature considered at ambient is *T* = 298*K* and the total pressure is assumed to be *P*_*total*_ = 1 atm = 1.01325 × 10^5^*Pa*.

The cardiac output is apportioned to the tissue compartments. All exchange is based on the perfusion limited model that assumes the tissue and venous blood are in equilibrium based on the partition coefficient for each compartment using Equation 3.

3 $$ \begin{array}{l}{C}_{venous}=\frac{C_{tissue}}{P{C}_{tissue: blood}}\\ {}\end{array} $$

where *C*_*tissue*_ is the concentration in the tissue for the compartment, and *PC*_*tissue:blood*_ is the partition coefficient for that tissue compartment and blood. The consequence of this assumption is that all exchanges between gas and blood, as well as blood and tissue compartments, are assumed to occur instantaneously. The resulting differential equation for the tissue compartment is

4 $$ \frac{d{C}_{tissue}}{dt}=\frac{Q_{tissue}\times \left({C}_{arterial}-\frac{C_{tissue}}{P{C}_{tissue: blood}}\right)}{V_{tissue}} $$

where *Q*_*tissue*_ and *V*_*tissue*_ are the perfusion and volume for the compartment, respectively, and *C*_*arterial*_ is the concentration in the arterial blood supplying the compartment.

One difference between our model and the Lockwood model follows from the definition of compartments; within Simbiology compartments must be defined in terms of volume, whereas Lockwood’s compartments are is in terms of mass [5]. Thus, assumed density values were used to derive the compartment volume fractions listed in Table 1. Other differences include the definition of organ compartments such as the brain from the richly perfused tissue compartment for some of our simulations as shown in Fig. 1.

The numerical solution of the model, a system of differential equations, was accomplished within Matlab using the “ode15s” code; a quasi-constant step size implementation in terms of backward differences of the Klopfenstein-Shampine family of numerical differentiation formulas [28, 29]. This method was efficient and stable, as no simulations took more than a minute on a computer workstation. A maximum step size of 0.5 s was used for numerical purposes, though we emphasize that the model results cannot be applied to this time resolution. A convergence test based the parameter area under the curve (AUC, to be explained below) resulted in a value within 0.01 % of the value for a time step of 0.1 s.

The physiological data for humans in Lockwood were extrapolated to pigs and rats based on data from several references [5, 12, 30–32]. The resulting parameters are compiled in Table 1. Note that detailed data such as a breakdown between rich and poorly perfused fat were not found for the other species.

Another key physical/chemical parameter is the specific partition coefficients for each gas in each compartment for each species. These data are not complete in the literature, thus requiring extrapolation from correlations from known data for other gases; for example, compartment:blood partition coefficients were determined from known fat:blood values using the linear correlations for organs described by Fiserova-Bergerova and Diaz [33]. We developed linear correlations for the vessel rich and vessel poor tissue compartment by making similar correlations using the data for six anesthetic gases analyzed in Lockwood [5]. For argon Ostwald solubility coefficients are available for blood, 0.037 [34], and for olive oil, 0.154 [35]. The solubility coefficient is equivalent to the partition coefficient if one of the compartments are in the gas phase [36]; in our model this is the case for the lung compartment. Furthermore, due to the scarcity of data, solubility in olive oil is used in lieu of fat [37]. The relevant fat:blood partition coefficient for argon is found by the ratio of (olive oil:gas)/(blood:gas), or 4.162.

The dose of a gas treatment is typically given as the concentration (molar, by volume, or by parts) of the inhaled gas, to the point that it is rare to find the use of classic PK parameters to assess the dose. Research based on inhaled dose as concentration of administered gas does not take into account complex physiological differences between species. Thus we use variables such as *C*_*max*_ the peak concentration for a particular compartment, *t*_*1/2*_, the time necessary to reduce the concentration from *C*_*max*_ by half, and *AUC* or the area under the curve, the integral of concentration through time that reflects the total exposure of the compartment to interpret the inhaled dose concentrations [38]. These data are derived (e.g., using numerical integration for *AUC*) from the discrete concentration data calculated at each time step for each compartment. *t*_*max*_, the time necessary to reach *C*_*max*_, is generally at the end of administration. However, due to a fast rise followed by slower uptake in fat, *t*_*max*_ is calculated as four times the exponential time constant estimated from the elimination time, *t*_*1/2*_.

The physiological parameters for each species are given in Table 1. The human is based on a 70 kg adult male as described by Lockwood [5]. Intersubject variability can be assessed by using scaling of compartment volumes, ventilatory parameters, and cardiac output and their distributions based on size, gender and age. For example, the percentage of fat as a function of body weight and gender is on average 13.5 % for men and 26.5 % for women [39]. It was assumed that the relative amounts of highly and poorly perfused fat and their perfusion were the same as the base human model [5]. Adaptations for arterial blood volume [40], cardiac output [41], and ventilatory parameters [42] were made based on correlations found in the literature.

## Results

**Fig. 2:**
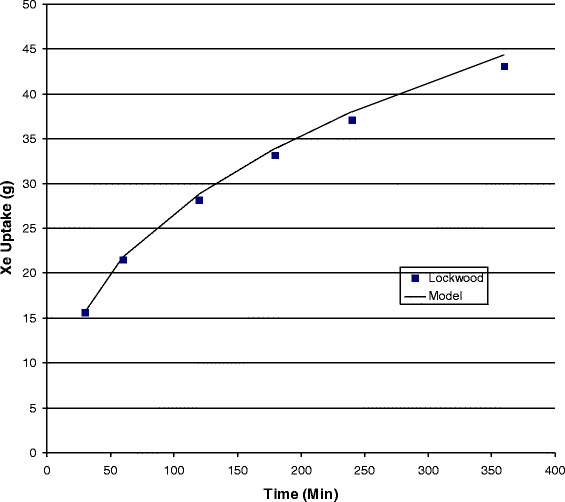
Comparison of xenon uptake in humans calculated using the current model to numerical data published by Lockwood [5]. This comparison between models is a validation of parameters and coding

**Fig. 3:**
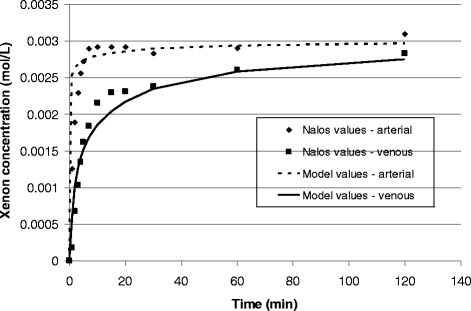
Comparison of xenon uptake in pigs calculated using the current model to experimental data published by Nalos et al. [43]. The Pearson regression coefficient r^2^ calculated in Excel (Microsoft, United States) for the models compared to the experimental data are 0.7923 and 0.9616 for the arterial and venous blood compartments, respectively

Validation is an important step in the use of any model. Unfortunately; *in vivo* measurements of noble gas concentration are difficult; and therefore, quite rare. The first comparison performed used data calculated by Lockwood [5] for xenon uptake as shown in Fig. 2. This is a validation of the parameters, system of equations, and solution techniques employed. The second validation comparison (shown in Fig. 3) was made from *in vivo* measurements of xenon concentration in arterial and mixed venous blood using gas chromatography-mass spectrometry of the head space gas over samples taken during the wash-in of xenon into eight pigs [43]. For this comparison compartment volumes are estimated because the weight of each pig was not available.

Pharmacokinetic results for exposure of 50 % xenon for one hour to an adult male, a pig and a rat are given in Table 2. Similar results are also given for 50 % argon for one hour in the human model.

**Table 2:** Pharmacokinetic results for a 60 min exposure to 50 % xenon or argon mixture with oxygen

Gas	*AUC* (mol.min/l)	*C*_*max*_ (mol/l)	*t*_*½*_ (min)	*t*_*max*_ (min)
Species				
Compartment				
Xenon Human				
Blood (venous)	1.31E-01	2.54E-03	3.64	42.0
Blood (arterial)	1.61E-01	2.78E-03	0.21	2.4
Liver	1.64E-01	2.98E-03	2.87	33.1
Muscle	1.10E-01	2.73E-03	15.83	182.7
Fat (Richly perfused)	1.66E-01	5.46E-03	29.12	336.1
Fat (Poorly perfused)	4.41E-02	1.49E-03	30.42	351.1
Richly perfused tissue	1.64E-01	2.94E-03	2.40	27.6
Poorly perfused tissue	1.58E-03	5.23E-04	29.44	339.8
Brain	1.73E-01	3.12E-03	2.52	29.1
Xenon Pig				
Blood (venous)	8.63E-02	1.77E-03	4.62	53.3
Blood (arterial)	1.26E-01	2.16E-03	0.19	2.2
Liver	1.67E-01	2.95E-03	1.59	18.4
Muscle	1.03E-01	2.61E-03	16.91	195.2
Fat	1.64E-01	5.37E-03	28.98	334.5
Richly perfused tissue	1.54E-01	2.97E-03	5.31	61.3
Poorly perfused tissue	8.42E-02	2.30E-03	20.40	235.4
Brain	1.37E-01	2.43E-03	1.79	20.7
Xenon Rat				
Blood (venous)	9.84E-01	3.41E-03	0.31	3.6
Blood (arterial)	1.20E + 00	4.05E-03	0.05	0.6
Liver	8.66E-01	2.93E-03	0.35	4.1
Muscle	2.46E-01	1.14E-03	2.29	26.4
Fat	1.52E + 00	7.15E-03	2.39	27.6
Richly perfused tissue	8.74E-01	2.94E-03	0.26	3.0
Brain	1.22E + 00	4.11E-03	0.09	1.0
Argon Human				
Blood (venous)	3.74E-02	7.02E-04	2.71	31.3
Blood (arterial)	4.41E-02	7.49E-04	0.18	2.1
Liver	3.21E-02	5.64E-04	1.89	21.8
Muscle	2.37E-02	5.26E-04	11.25	129.9
Fat (Richly perfused)	4.18E-02	1.30E-03	26.55	306.4
Fat (Poorly perfused)	1.19E-02	3.93E-04	29.48	340.3
Richly perfused tissue	4.36E-02	7.70E-04	2.07	23.9
Poorly perfused tissue	4.35E-03	1.42E-04	28.93	333.8
Brain	2.90E-02	5.05E-04	1.46	16.8

**Fig. 4:**
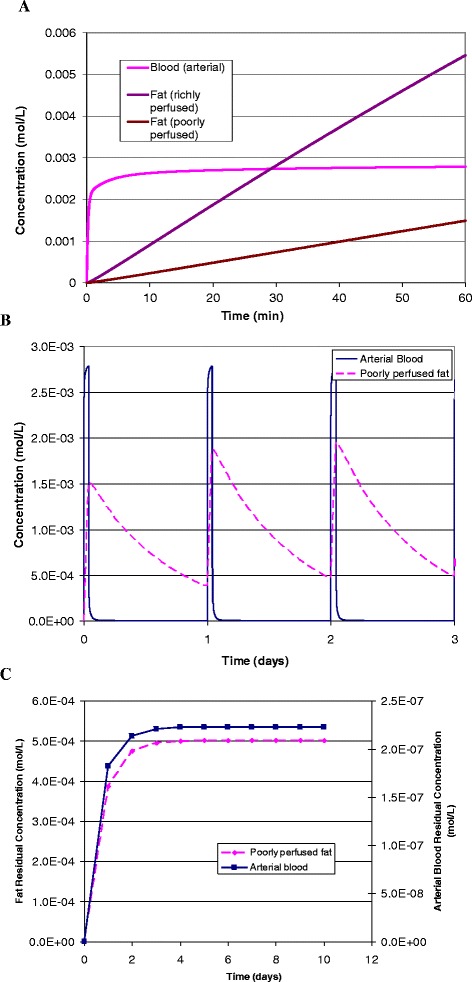
**a**. Arterial blood, richly and poorly perfused fat xenon concentrations after a single administration for 1 hr of 50 % xenon to a male adult human. **b**. Poorly perfused fat xenon concentration after the repeated administration of the same dose once per day. **c**. Residual xenon concentration just before the next administration for 10 days. The residual dose is at a relatively low level (equivalent to breathing 0.01 % xenon gas composition) but this is still about a thousand times greater than ambient exposure

Graphically, the results of a one hour exposure to xenon are given in Fig. 4a in terms of arterial blood concentration and xenon concentration in the two fat compartments that have different perfusion rates. The very different timing of absorption rates is evident between the compartments. These differences are also apparent in the example of chronic, or repeated, dosing shown in Fig. 4b. In this case the one-hour exposure to 50 % xenon is repeated once per day for 10 days. Note that the peak concentration and the residual concentration, the concentration just before the next exposure, are rising in the fat compartment over the three days shown in the figure. There are no apparent differences each day in the arterial blood concentration. However, in Fig. 4c residual concentrations for arterial blood and poorly perfused fat are shown using different scales, where it is clear that the residual concentration in both compartments increases for about 5 days before reaching a plateau.

**Fig. 5:**
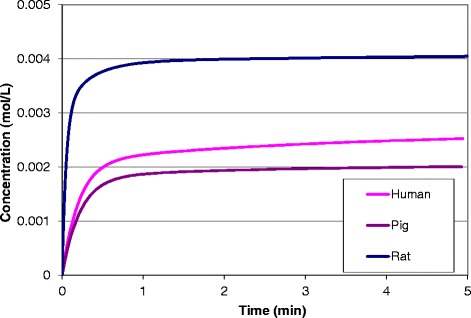
Comparison of arterial blood xenon concentration after a single administration of 1 hr of 50 % xenon for a small animal (rat), a large animal, (pig), and a male adult human

In Fig. 5 are shown the plots of arterial blood concentration during exposure to 50 % xenon in the adult human male, rat (the mouse, not shown for clarity, is close to the rat) and pig models. The human and pig are similar while the concentration in the rat is almost double. Also note that the timing is almost ten times faster in the rat than in the larger species as reflected in the data given in Table 2.

**Fig. 6:**
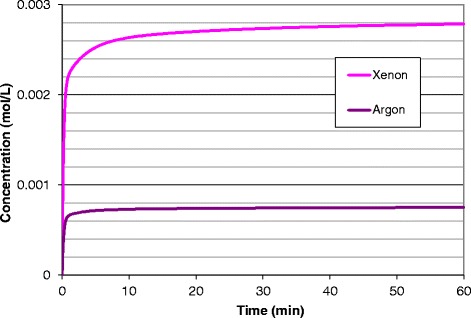
Variation of uptake with gas type; comparison of arterial blood concentration after a single administration of 1 hr of 50 % xenon or argon to a male adult human

Results from simulations of one-hour exposures to 50 % xenon and argon in the human model are shown in Fig. 6 in the form of arterial blood concentrations as a function of time. Note, that the equivalent delivered doses of 50 % for one hour result in very different pharmacokinetic doses (e.g., see the *AUC* for the brain for these two cases in Table 2: 0.173 for xenon and 0.029 for argon).

**Fig. 7:**
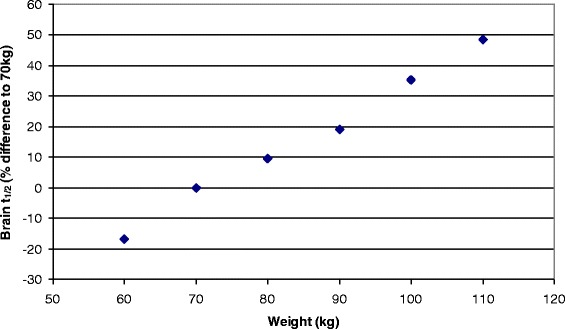
An example of intersubject variability; the variation of *t*_*1/2*_ for the brain compartment for administration of xenon as a function of weight for adult males. The variation is expressed as a percentage of the value for a 70 kg individual

An example of the effect of intersubject variability on pharmacokinetics of gases is shown in Fig. 7. We note that in general, *Cmax* is determined by the partition coefficients, such that there is very little intersubject variability for this parameter. However, the kinetics are a function of the relative volume distribution between the compartments. Thus, there is significant variation in *t*_*1/2*_ as a function of weight as shown in this plot of for adult males who have received a 50 % exposure to xenon for one hour.

## Discussion

A pharmacokinetic model has been presented for the chronic administration with repeated dosing of inert, noble gases (xenon and argon) to humans, pigs and rats. The absorption, distribution, metabolism and excretion (ADME) modeled are very simple; absorption in all compartments is assumed to be perfusion limited, the gases are not metabolized, and excretion is only by the reverse process of absorption through the lungs and exhaled.

The model was validated by comparison to published data; to xenon uptake data in humans from a different model [5] and from experimental measurements of xenon blood concentrations during wash-in to pigs [43]. Both comparisons (Figs. 2 and 3) show that the current model can accurately determine the rate of disposition of the gas in the body. We note that this accuracy is achieved in spite of the fact that the model does not account for mixing or circulation in the arterial blood compartment.

Based on the example of a one-hour administration of xenon per day, the key physiological results show that fat acts as a reservoir for gas storage, such that there is a residual dose that is equivalent to a continuous exposure. The residual dose, the concentration just before the re-administration, is at a relatively low level (equivalent to breathing 0.01 % xenon gas composition) but this is still about a thousand times greater than ambient exposure (8.7x10^-6^ % of xenon in the atmosphere) in humans. It is important for preclinical testing that the clearance is much faster in small animals (about 10 times faster, see *t*_*1/2*_ for rats in Table 2) such that there is essentially no residual dose between exposures for the same chronic application example presented for humans in Fig. 4b and c. Thus, preclinical studies with small animals might not indicate benefits or the negative effects associated with these therapies that occur after administration of the gas. However, preclinical studies could be designed to include a residual dose to mimic the effect of gas storage in fatty tissue that occurs for humans. Note that argon differs from xenon in that the residual dose is not important because the baseline atmospheric concentration is 1 %.

Another important aspect of interspecies variability is the different tissue solubility for each gas among animal species as indicated by the partition coefficients listed in Table 1 and the arterial blood concentration plots shown in Fig. 5. Furthermore, these differences are expressed in the differences in dose for each species in each compartment listed in Table 2. The key message is that the dose delivered to the patient, in this example 50 % xenon for one hour, will be different at the site of action for each species. Changes in concentration can readily be implemented using Equation 2, including hyperbaric conditions. The most important and direct effect of changes in concentration are proportional changes in saturation concentrations.

To expand on the point of characterizing experiments with the true dose (i.e., the *AUC* at the site of action) the gas delivery system must be considered. Due to the physics related to filling the dead volume or leaks in the delivery system it is virtually impossible to immediately apply the inhaled dose as has been modeled herein. Thus the wash-in of gas into animal boxes [44] and ventilators [45] should be taken into account to determine the true *AUC* and is the subject of future work for our team.

The measure of intersubject variability in this ADME PBPK model only accounts for physiological parameters, it does not take into account pharmacological variations that are also known to occur due to age and gender (e.g., for xenon anesthesia [46]).

There are several aspects of the model that can be improved. The basic, steady state model for respiration can be improved to better account for the complex distribution of gas concentration that exists in real lungs (for example oxygen [27] and nitric oxide [47] uptake have been examined in the literature). There may be exceptions to the perfusion limited assumption used in the model. For example, studies in volunteers have shown non-uniform distributions of xenon in blood as a function of hemocrit [48] and in the brain [49] using computed tomography imaging. Another study in sheep [50] has shown that a PK model with direct diffusion between brain regions was better for fitting the experimental data for the absorption of helium. In principle, the model can readily be extended to other gases where metabolism does not occur or is insignificant, krypton, neon, helium, nitrous oxide, and nitrogen; the last use of the model would be to investigate the di-nitrogenation process that is necessary to optimize the delivery of gases using recirculating systems [45]. However, all the necessary partition coefficients are not readily available in the literature for all compartments, animal species, and gases. Certainly, more experimental data of pharmacokinetics with pharmacodynamics are needed for the development of optimized gas therapies.

## Conclusions

An ADME PBPK model has been developed to investigate the unique aspects of the chronic administration of noble gas therapies. The model has shown that there can be a residual dose, equivalent to constant administration, for chronic repeated dosing of xenon in humans. However, this is not necessarily the case for small animals used in pre-clinical studies. The use of standard PK parameters such as *AUC* would be more appropriate to assess the delivered dose than the gas concentration in the delivery system that is typically cited.

## Abbreviations

ADME: Absorption, distribution, metabolism and excretion
AUC: Area under the curve
Bax: Bcl-2-associated X protein
Bcl-2/Bcl-xL: B-cell lymphoma 2/ B-cell lymphoma-extra large
ERK_1/2_: Extracellular-signal-regulated kinases 1/2
MAPK: Mitogen-activated protein kinase
NMDA/AMPA: N-methyl-D-aspartate, /α-Amino-3-hydroxy-5-methyl-4-isoxazolepropionic acid
p38: p38 is in fact p38 MAPK and means Mitogen-Activated Protein Kinase
PK: Pharmacokinetics
PB: Physiological Based
TREK-1: TWIK-1 related K+ channel-1

## References

[1] Zhang JH (2011). Welcome to medical gas research. Med Gas Res.

[2] Esencan E, Yuksel S, Tosun YB, Robinot A, Solaroglu I, Zhang JH (2013). XENON in medical area: emphasis on neuroprotection in hypoxia and anesthesia. Med Gas Res.

[3] Liu W, Liu Y, Chen H, Liu K, Tao H, Sun X (2013). Xenon preconditioning: molecular mechanisms and biological effects. Med Gas Res.

[4] Coburn M, Sanders RD, Ma D, Fries M, Rex S, Magalon G (2012). Argon: the ‘lazy’ noble gas with organoprotective properties. Eur J Anaesthesiol.

[5] Lockwood G (2010). Theoretical context-sensitive elimination times for inhalation anaesthetics. Br J Anaesth.

[6] Smith MA, Sapsed-Byrne SM, Lockwood GG (1997). A new method for measurement of anaesthetic partial pressure in blood. Br J Anaesth.

[7] Bolt HM, Filser JG, Buchter A (1981). Inhalation pharmacokinetics based on gas uptake studies. III. A pharmacokinetic assessment in man of “peak concentrations” of vinyl chloride. Arch Toxicol.

[8] Bolt HM, Filser JG, Stormer F (1984). Inhalation pharmacokinetics based on gas uptake studies. V. Comparative pharmacokinetics of ethylene and 1,3-butadiene in rats. Arch Toxicol.

[9] Filser JG, Bolt HM (1981). Inhalation pharmacokinetics based on gas uptake studies. I. Improvement of kinetic models. Arch Toxicol.

[10] Filser JG, Bolt HM (1983). Inhalation pharmacokinetics based on gas uptake studies. IV. The endogenous production of volatile compounds. Arch Toxicol.

[11] Filser JG, Bolt HM (1984). Inhalation pharmacokinetics based on gas uptake studies. VI. Comparative evaluation of ethylene oxide and butadiene monoxide as exhaled reactive metabolites of ethylene and 1, 3-butadiene in rats. Arch Toxicol.

[12] Filser JG, Schmidbauer R, Rampf F, Baur CM, Putz P, Csanady GA (2000). Toxicokinetics of inhaled propylene in mouse, rat, and human. Toxicol Appl Pharmacol.

[13] Deng J, Lei C, Chen Y, Fang Z, Yang Q, Zhang H (2014). Neuroprotective gases-fantasy or reality for clinical use?. Prog Neurobiol.

[14] Haseneder R, Kratzer S, Kochs E, Mattusch C, Eder M, Rammes G (2009). Xenon attenuates excitatory synaptic transmission in the rodent prefrontal cortex and spinal cord dorsal horn. Anesthesiology.

[15] Spaggiari S, Kepp O, Rello-Varona S, Chaba K, Adjemian S, Pype J (2013). Antiapoptotic activity of argon and xenon. Cell Cycle.

[16] Zhuang L, Yang T, Zhao H, Fidalgo ANR, Vizcaychipi MP, Sanders RD (2012). The protective profile of argon, helium, and xenon in a model of neonatal asphyxia in rats. Crit Care Med.

[17] Weber NC, Toma O, Wolter JI, Obal D, Müllenheim J, Preckel B (2005). The noble gas xenon induces pharmacological preconditioning in the rat heart *in vivo* via induction of PKC-epsilon and p38 MAPK. Br J Pharmacol.

[18] Fahlenkamp AV, Rossaint R, Haase H, Kassam HA, Ryang YM, Beyer C (2012). The noble gas argon modifies extracellular signal-regulated kinase 1/2 signaling in neurons and glial cells. Eur J Pharmacol.

[19] Bantel C, Maze M, Trapp S (2009). Neuronal preconditioning by inhalational anesthetics: evidence for the role of plasmalemmal adenosine triphosphate-sensitive potassium channels. Anesthesiology.

[20] Gruss M, Bushell TJ, Bright DP, Lieb WR, Mathie A, Franks NP (2004). Two-pore-domain K+ channels are a novel target for the anesthetic gases xenon, nitrous oxide, and cyclopropane. Mol Pharm.

[21] Coburn M, Sanders RD, Ma D, Fries M, Rex S, Magalon G (2012). Argon: the ‘lazy’ noble gas with organo protective properties. Eur J Anaesthesiol.

[22] Bessiere B, Laboureyras E, Laulin JP, Simonnet G (2010). Xenon prevents inflammation-induced delayed pain hypersensitivity in rats. Neuroreport.

[23] Vengeliene V, Bessiere B, Pype J, Spanagel R (2014). The Effects of xenon and nitrous oxide gases on alcohol relapse. Alcohol Clin Exp Res.

[24] Tawhai MH, Pullan AJ, Hunter PJ (2000). Generation of an anatomically based three-dimensional model of the conducting airways. Ann Biomed Eng.

[25] Tawhai MH, Hunter PJ (2001). Characterising respiratory airway gas mixing using a lumped parameter model of the pulmonary acinus. Respir Physiol.

[26] Politi AZ, Donovan GM, Tawhai MH, Sanderson MJ, Lauzon AM, Bates JH (2010). A multiscale, spatially distributed model of asthmatic airway hyper-responsiveness. J Theor Biol.

[27] Foucquier A, Filoche M, Moreira AA, Andrade JS, Arbia G, Sapoval B (2013). A first principles calculation of the oxygen uptake in the human pulmonary acinus at maximal exercise. Respir Physiol Neurobiol.

[28] Shampine LF, Reichelt MW (1997). The MATLAB ODE suite, SIAM. J Sci Comp.

[29] Shampine LF, Reichelt MW (1999). Solving index-1 DAEs in MATLAB and simulink, SIAM. Review.

[30] Csanady GA, Filser JG (2007). A physiological toxicokinetic model for inhaled propylene oxide in rat and human with special emphasis on the nose. Toxicol Sci.

[31] Fiserova-Bergerova V (1992). Inhalation anesthesia using physiologically based pharmacokinetic models. Drug Metab Rev.

[32] Fiserova-Bergerova V (1995). Extrapolation of physiological parameters for physiologically based simulation models. Toxicol Lett.

[33] Fiserova-Bergerova V, Dingley J (1986). Determination and prediction of tissue-gas partition coefficients. Int Arch Occup Environ Health.

[34] Rosenthal MS, Nickles RJ (1985). Selected noble-gas partition coefficients. Phys Med Biol.

[35] Veall N, Mallett BL (1965). The partition of trace amounts of xenon between human blood and brain tissues at 37 C. Phys Med Biol.

[36] Florence AT, Attwood D. Physicochemical principles of pharmacy. Pharmaceutical Press, 2011.

[37] Van Rees H (1974). The partition coefficients of styrene between blood and air and between oil and blood. Int Arch Arbeitsmed.

[38] Rowland M, Tozer TN (1995). Clinical pharmacokinetics: Concepts and applications.

[39] Jeukendrup A, Gleeson M. Sport nutrition: an introduction to energy production and performance. Human Kinetics, 2010.

[40] Sackner MA, Atkins N, Goldberg J, Segel N, Zarzecki S, Wanner A (1974). Pulmonary arterial blood volume and tissue volume in man and dog. Circ Res.

[41] Collis T, Devereux RB, Roman MJ, de Simone G, Yeh JL, Howard BV (2001). Relations of stroke volume and cardiac output to body composition The Strong Heart Study. Circulation.

[42] Bailey MR (1994). The new ICRP model for the respiratory tract. Radiat Prot Dosim.

[43] Nalos M, Wachter U, Pittner A, Georgieff M, Radermacher P, Froeba G (2001). Arterial and mixed venous xenon blood concentrations in pigs during wash-in of inhalational anaesthesia. Br J Anaesth.

[44] Leavens TL, Moss OR, Bond JA (1996). Dynamic inhalation system for individual whole-body exposure of mice to volatile organic chemicals. Inhal Tox.

[45] Rawat S, Dingley J (2010). Closed-circuit xenon delivery using a standard anesthesia workstation. Anesth Analg.

[46] Goto T, Nakata Y, Morita S (2002). The minimum alveolar concentration of xenon in the elderly is sex-dependent. Anesthesiology.

[47] Martin A, Jackson C, Katz I, Caillibotte G (2014). Variability in uptake efficiency for pulsed versus constant concentration delivery of inhaled nitric oxide. Med Gas Res.

[48] Sesay M, Kolanek B, Pena D, Tauzin-Fin P, Dousset V, Nouette-Gaulain K. Effet de la variation du taux d’hématocrite sur le coefficient de partage cerveau-sang du Xénon mesuré par tomodensitométrie. In *Annales Françaises d’Anesthésie et de Réanimation*, vol. 32, Elsevier Masson, 2013, p. A321-A322.

[49] Sesay M, Nouette-Gaulain K, Tauzin-Fin P, Vincent D, Maurette P. Evaluation of the brain-blood partition coefficient of xenon in healthy volunteers. Proceedings of the 2010 Annual Meeting of the American Society Anesthesiologists, October 16 - 20, San Diego, California. 2010. http://www.asa-abstracts.com/strands/asaabstracts/abstract.htm;jsessionid=4AEB68C4387ACBE4A5BA4E5F664E03FD?year=2010&index=15&absnum=1370.

[50] Doolette DJ, Upton RN, Grant C (2005). Perfusion − diffusion compartmental models describe cerebral helium kinetics at high and low cerebral blood flows in sheep. J Physiol.

